# National Hospital for the Paralysed

**Published:** 1905-06-24

**Authors:** 


### NATIONAL HOSPITAL FOR THE PARALYSED.

Lord Strathcona and Mount Royal presided at the festival dinner of the National Hospital for the Paralysed and Epileptic, which was held on the 15th inst. at the Whitehall Rooms with the object of meeting the deficit between the ordinary income and the ordinary expenditure which in 1904 amounted to £5,041. Amongst those present were Viscount Hampton, Sir Felix Semon, C.V.O., the Hon. J. H. and Mrs. Turner, the Dean of Canterbury, Mr. Frederick Macmillan (Chairman of the Board of Management), Dr. David Ferrier and Mrs. Ferrier, Hon. Alfred Dobson, C.M.G., Mr. J. Danvers Power, and Dr. J. A. Ormerod.

The Chairman, in proposing "Success to the National Hospital for the Paralysed and Epileptic," said that the hospital had a reputation not only in London, not only in the United Kingdom, not only in the British Empire, but throughout the whole world, for patients came to it from all parts. Besides this, medical men—physicians and surgeons—came to it, also, from all over the globe, making it a post-graduation institution, to learn what they had not the same opportunities of learning in their own countries. The convalescent home in connection with the hospital, had of late, unfortunately for want of funds, only been partially open to free patients, and they now admitted paying patients. This state of things, however, the Committee hoped to remedy in the near future.

Mr. Frederick Macmillan responded. He said that within the last year or two a very careful scrutiny had been made of the expenses of the hospital, with the result that they could now promise their subscribers that for every pound they gave they would have twenty shillings' worth of good. There could not now, he felt sure, be any further economies made as the board of management did not intend to sacrifice efficiency to economy. The diseases which were treated at the National Hospital were of an obscure and distressing character, and appeared under the conditions of modern life to be increasing in virulence and frequency. The National Hospital was by far the most important institution which dealt with those kinds of disease, and its work was not by any means confined to the City of London, or even to the United Kingdom, but treatment was given to patients who came from the uttermost parts of the earth. The great ne[illegible] of the hospital at the present time was more annual su[illegible] scribers.

Dr. Ormerod, in replying for the "Medical Staff" (proposed by Mr. J. Danvers Power), referred to what the Chairman had mentioned as to the hospital being used by medical men as a post-graduation institution, and said that during 1904 besides the number of medical men who had attended from the United Kingdom, 84 had attended at the hospital from Canada, 86 from the United States, 20 from France, besides others who had come from Australia, New Zealand, Cape Colony, India, Ceylon, Peru, Venezuela, Austria, Switzerland, Italy, Germany, Denmark, Norway, Bulgaria, Egypt, and Persia.

The Secretary announced that a sum of £3,077 had been collected as the result of the dinner.

